# Impact of Diglossia on Word and Non-word Repetition among Language Impaired and Typically Developing Arabic Native Speaking Children

**DOI:** 10.3389/fpsyg.2017.02010

**Published:** 2017-11-22

**Authors:** Elinor Saiegh-Haddad, Ola Ghawi-Dakwar

**Affiliations:** ^1^English Linguistics and Literature Department, Bar-Ilan University, Ramat Gan, Israel; ^2^Sakhnin College for Education, Ministry of Education, Tel Aviv, Israel

**Keywords:** Arabic, specific language impairment (SLI), language disorders, diglossia, non-word repetiton, linguistic distance, pseudo word learning

## Abstract

The study tested the impact of the phonological and lexical distance between a dialect of *Palestinian Arabic* spoken in the north of Israel (SpA) and Modern Standard Arabic (StA or MSA) on word and non-word repetition in children with specific language impairment (SLI) and in typically developing (TD) age-matched controls. Fifty kindergarten children (25 SLI, 25 TD; mean age 5;5) and fifty first grade children (25 SLI, 25 TD; mean age 6:11) were tested with a repetition task for 1–4 syllable long real words and pseudo words; Items varied systematically in whether each encoded a novel StA phoneme or not, namely a phoneme that is only used in StA but not in the spoken dialect targeted. Real words also varied in whether they were lexically novel, meaning whether the word is used only in StA, but not in SpA. SLI children were found to significantly underperform TD children on all repetition tasks indicating a general phonological memory deficit. More interesting for the current investigation is the observed strong and consistent effect of phonological novelty on word and non-word repetition in SLI and TD children, with a stronger effect observed in SLI. In contrast with phonological novelty, the effect of lexical novelty on word repetition was limited and it did not interact with group. The results are argued to reflect the role of linguistic distance in phonological memory for novel linguistic units in Arabic SLI and, hence, to support a specific *Linguistic Distance Hypothesis* of SLI in a diglossic setting. The implications of the findings for assessment, diagnosis and intervention with Arabic speaking children with SLI are discussed.

## Introduction

Specific language impairment (hereafter, SLI; also referred to as Language Disorder, LD) affects ≈3.5–7% of the children (Tomblin et al., 1996) and is defined as “persistent difficulties in the acquisition and use of language… [when] the difficulties are not attributable to hearing or other sensory impairment, motor dysfunction, or another medical or neurological condition, and are not better explained by intellectual disability or global developmental delay” (American Psychiatric Association, 2013, p. 42). SLI can have a variegated phenotype and children with SLI demonstrate very heterogeneous profiles (Leonard, 1998), including lower than expected for-their-age vocabulary: expressive and receptive and grammar: basic and complex (Leonard and Bortolini, 1998; Dromi et al., 1999; Bedore and Leonard, 2001; Stavrakaki, 2001; Friedmann and Novogrodsky, 2004, 2007, 2011; Marshall et al., 2007; Penke, 2009; van der Lely et al., 2011). SLI children also reveal remarkable phonological deficits when compared with their age-matched controls, including deficits in auditory phonological processing and memory (Gathercole and Baddeley, 1990; Tallal et al., 1991, 1993; Joanisse and Seidenberg, 1998; Newbury et al., 2005), phonological representations and awareness (Thatcher, 2010; Claessen and Leitão, 2012; Rispens and Baker, 2012), phonological decoding in word reading (Conti-Ramsden and Durkin, 2007; Tambyraja et al., 2015), and phonological learning.

### Word repetition deficits in SLI

Given a variegated phenotype, several theories were proposed to capture the etiology of SLI. According to one theory, SLI results from a deficit in input processing capacity, such as phonological short-term memory (Gathercole and Baddeley, 1990) or auditory or phonological processing (Tallal et al., 1991, 1993; Joanisse and Seidenberg, 1998). This theory predicts that impaired phonological processing in SLI will result in word/non-word repetition deficits, especially when the repetition tasks target long words and non-words. This is because repetition, especially of long items mostly targeted by earlier research, requires the temporary storage and processing of phonological information in memory^1^. This hypothesis received strong support in the finding that children with language impairment performed significantly more poorly than their age-matched typically developing peers on repetition tasks (Gathercole and Baddeley, 1990; Montgomery, 1995; Dollaghan and Campbell, 1998; Edwards and Lahey, 1998; Weismer et al., 2000; Newbury et al., 2005). The question that follows from this finding, however, pertains to the specific nature of the repetition deficit. Namely, the specific phonological skills implicated in word repetition, and the extent to which it might be influenced by linguistic structural factors (such as phonotactic probabilities, morphological structure, etc.) *vis-a-vis* functional sociolinguistic factors (such as spheres of use, experience, practice, etc.). Both sets of factors are expected to impact phonological processing in memory and might, thus, be associated with intrapersonal and inter-personal differences in repetition capacities.

Research on word/non-word repetition task performance has thus far focused primarily on structural linguistic factors to the exclusion of sociolinguistic functional factors. This research endeavor has shown that language-specific linguistic factors, such as phonotactic probability, syllabic length, and phonological similarity with real words influence repetition performance (Gathercole and Baddeley, 1990; Gathercole, 1995, 2006). Moreover, it has been shown that SLI and TD children may vary in degree of sensitivity to these factors, with SLI children's repetition being more vulnerable to linguistic manipulations affecting the word-likeness of stimuli (Munson et al., 2005; Graf Estes et al., 2007; Armon-Lotem and Chiat, 2012), probably because functional exposure to input, which is critical for constructing proper phonological representations, is limited in SLI (Armon-Lotem, 2017). These findings imply that word and non-word repetition tasks are not free of lexical influences and might implicate lexical factors such as linguistic representations stored in long-term memory. In turn, the repetition deficits observed in SLI might not reflect phonological memory storage and processing deficits only, but also impaired or low-quality (e.g., inaccurate, fuzzy, unstable) lexical representations (Swan and Goswami, 1997; Perfetti, 2007).

Several researchers have argued that deficits in phonological processing in working memory may couch in difficulties in establishing, accessing, and retrieving phonological representations from long-term memory (Gathercole and Adams, 1993; Dollaghan and Campbell, 1995; Weismer et al., 2000; Sutherland and Gillon, 2007; Pennington and Bishop, 2009; Claessen and Leitão, 2012; Rispens and Baker, 2012). Evidence supporting this position comes partly from research showing that repetition of non-words, especially wordlike non-words, is correlated with vocabulary size, though the nature of the relationship between the two abilities is yet unclear (Gathercole and Baddeley, 1989; Service, 1992; Service and Kohonen, 1995; Dufva and Voeten, 1999; Masoura and Gathercole, 1999; Metsala, 1999; Conti-Ramsden, 2003; Gathercole, 2006; Hoff et al., 2008; Rispens and Baker, 2012; Engel de Abreu et al., 2013). Relatedly, it has been shown that non-word repetition is influenced by the wordlikeness of items (Gathercole and Baddeley, 1990; Dollaghan and Campbell, 1995; Gathercole, 1995, 2006; Munson et al., 2005). These effects suggest that phonological processing in working memory is impacted by knowledge stored in long term memory, and are in keeping with Baddeley's (2003) multi-componential model of working memory. These effects are also in keeping with the *Lexical Restructuring Model*, which captures the development of phonological representations in the lexicon of typically developing children and suggests a positive influence of growth in vocabulary size on phonological representational quality and, in turn, on phonological processing (Metsala, 1997a,b, 1999; Metsala and Walley, 1998).

*Word-likeness* (Gathercole, 1995), as a phonological property of non-words, has thus far been operationalized mainly in terms of the compositional phonological structure of non-word items, and the extent to which this structure abides by the linguistic patterns of the language under question (e.g., phonotactic probabilities, morphological structure, stress, etc.). Thus, word-likeness has not yet been operationalized in any systematic way in terms of the identities of the phonological structures within the non-word items; specifically of whether items depict novel structures that are not within the spoken linguistic repertoire of children. In effect, it has mostly been agnostic of variations in extent of experience and practice with specific phonological structures, and the effect of this factor on repetition capacity. Gibson et al. (2015) addressed the role of language experience on repetition capacity among Spanish-dominant and English-dominant Spanish–English bilingual 5-year-old children. They found the Spanish-dominant group performed better than the English-dominant group for both Spanish and English non-words. Moreover, Spanish non-words were produced more accurately than English non-words overall. These findings were argued to reflect the extra practice the dominant Spanish speakers had with producing multisyllabic words.

Arabic diglossia offers another natural setting in which within-subject variations in extent of language experience on repetition ability may be tested. This is because native speakers in Arabic diglossia, and even the young ones among them, acquire two linguistic systems for two complementary sets of social functions: one for everyday speech and another for formal speech and writing. As a result, for most of the words they know, Arabic speakers store two phonological forms: one spoken/colloquial and another standard/written. Moreover, the two forms of many words in their lexicons may vary in just one constituent phoneme, with the standard word embodying a standard novel phoneme that is not within the spoken variety of children. This property can be authentically manipulated in constructing word and non-word repetition tasks in order to shed light on the role of this specific phonological feature on repetition ability. Furthermore, manipulating phonemic novelty, as a sociolinguistically based factor, in the selection of Arabic words, and in the construction of non-words, allows an investigation of whether SLI and TD children are equally affected by this factor. This question will have important implications for the nature of the phonological constraints on repetition ability, as well as the nature of the underlying phonological deficit in SLI, and its susceptibility to language experience.

Relatedly, in Arabic diglossia, it is possible to tease apart phonological novelty from lexical novelty. Because words may have two different phonological forms, the lexical store of Arabic speaking children may be broken down into four types of words: (a) lexically and phonologically non-novel, (b) lexically non-novel but phonologically novel, (c) lexically novel but phonologically non-novel, and (d) lexically and phonologically novel (Saiegh-Haddad, 2004; Saiegh-Haddad and Spolsky, 2014). In turn, it is possible to test the independent contribution of lexical and phonological novelty to word repetition. Furthermore, in the case of non-words, it is possible to test non-word repetition not only for non-words whose compositional phonemic form is novel, namely they string together non-novel phonemes in a novel order (which is how non-words are usually created) but also for non-words whose internal phoneme(s) are novel. This will allow an examination of the independent effect of these two aspects of phonological novelty on word repetition in TD and in SLI children.

### Diglossia: impact on language processing

Arabic is a prototypical case of the concept diglossia as it was first outlined by Ferguson (1959): “a relatively stable language situation in which, in addition to the primary dialects of the language (which may include a standard or regional standards), there is a very divergent, highly codified (often grammatically more complex) superposed variety …. which is learned largely by formal education and is used for most written and formal spoken purposes but is not used by any section of the community for ordinary conversation” (p. 336). Ordinary everyday conversation in Arabic is conducted using a specific local spoken vernacular, collectively referred to as Spoken Arabic (or Colloquial Arabic). This variety is acquired naturally as a mother tongue. In contrast, the modern standard variety: Modern Standard Arabic (StA) is the language of conventional literacy tasks (reading and writing), as well as formal speech, and is learnt mainly in the formal classroom setting with special focus on grammatical accuracy; It is a modern descendant of Classical Arabic and of Literary Arabic and is to a high degree uniform across the Arabic speaking world.

Hence, in all regions in the Arabic-speaking world, once children enter school they are intensively and extensively exposed to Modern Standard Arabic as the language of reading and writing. Spoken interactions, even inside the classroom, remain to be conducted in Spoken Arabic, or in a semi-standard variety known as *Educated Spoken Arabic* (Badawi, 1973), except probably during Arabic lessons, where Standard Arabic is more dominant, at least in aspiration (Amara, 1995). The great majority of Arabic speaking Palestinian citizens of Israel are native speakers of Arabic and the great majority of them enroll in Arabic-medium schools (preschool throughout high-school). In these schools, Arabic is the only language of instruction and textbooks, and all school subjects are taught exclusively in Arabic, including math and science. Hebrew and English are both taught as second/foreign languages starting in the third and fourth grades, respectively (For more, see Saiegh-Haddad and Everatt, 2017).

Despite such deceivingly dichotomous context, and while Spoken Arabic is undoubtedly the primary spoken language, native speakers of Arabic, including young children, are actively and constantly engaged with Standard Arabic as well; they pray, do their homework and study for their exams in Standard Arabic, and they also watch many TV programs and dubbed series in this variety. Thus, besides proficiency in using Spoken Arabic, linguistic development in Arabic involves, from an early age, concurrent acquisition of Standard Arabic^2^.

Because StA is the language of formal speech and reading/writing, it permeates the speech of many speakers, and this dynamic infusion happens in all linguistic domains (phonology, syntax, morphology, lexicon). As a result, it is often difficult to draw clear boundaries between the spoken and written norms. In fact, though Ferguson proposes a dichotomy between the spoken and written varieties, he himself recognizes that this is just an abstraction. Rather, the complex linguistic situation in Arabic diglossia has been described in terms of levels, or even a continuum, with speakers shifting between what may be conceived of as an infinite number of varieties (Bassiouny, 2009) ranging between colloquial/vernacular and literary/standard forms (Blanc, 1960; Badawi, 1973; Meiseles, 1980; Boussofara-Omar, 2006).

A conspicuous feature of Arabic diglossia is a phonological and a lexical distance between Standard Arabic and Spoken Arabic (for a comprehensive discussion, see Saiegh-Haddad and Henkin-Roitfarb, 2014). This distance might take different forms in different Arabic-speaking regions. Yet, no Spoken vernacular shares the exact set of phonemes, or the same set of lexical items with Standard Arabic (Maamouri, 1998). For instance, in the domain of phonology, Standard Arabic comprises 28 consonantal phonemes and six vowel phonemes: three short vowels: low /*a*/, high front /*i*/, and high back /*u*/, and three corresponding long vowels: /*a:*/, /i:/, and /*u:*/. Moreover, all syllables in Standard Arabic must begin with a single consonant (C) serving as the syllable onset and followed by a vowel (V), as the syllable nucleus/peak. Yet, this phonological structure is at variance with that of many varieties of Spoken Arabic which usually comprise a smaller set of consonants and a larger set of vowels. To illustrate, interdental consonants are not within the phonemic inventory of many dialects of *Palestinian Arabic* spoken in the north of Israel. As a result, *Cognate words*, which are StA words that are also used in these dialects, acquire a different phonological form than that used in StA with StA interdental phonemes substituted for by corresponding phonemes used in these varieties of Spoken Arabic (StA /*ӨaɁlab*/; SpA /*taɁlab*/ “fox”). Similarly, the glottal stop phoneme, especially when preceded by a long vowel, is not preferred in a word-final position in these dialects. Therefore, cognate words ending in a glottal stop often delete this phoneme and reduce the preceding vowel (StA /*sama:*Ɂ/; SpA /*sama*/ “sky”). Finally, consonantal cluster codas, which are widespread in monosyllabic StA words (in *pausal* non-inflected form) are not preferred in these dialects and, therefore, such clusters are usually broken through the insertion of an epenthetic vowel (StA /*ba*ħ*r*/; SpA /*ba*ħ*ir*/ or /*bahar*/ “sea”).

The lexical distance between Standard and Spoken Arabic is pervasive. To assess the scope of this distance, Saiegh-Haddad and Spolsky (2014) analyzed a corpus of 4,500 word-types derived from a pool of 17,500 word-tokens collected from 5-year-old native speakers of a local dialect of *Palestinian Arabic* spoken in the center of Israel. This study showed that only 21.2% of the words in the child's spoken lexicon were *Identical words*, that is words that keep an identical lexico-phonological form in SpA and StA (e.g., /*na:m*/ “slept”; /*daftar*/ “notebook”), whereas the remaining words were approximately evenly divided between *Cognate words*, which are shared by the two varieties, yet keep partially overlapping phonological forms in each of them (e.g., SpA /*dahab* /vs. StA /ð*ahab*/ “gold”), and *Unique SpA words*, which have a unique lexico-phonological form in SpA completely different from its form in StA (e.g., SpA /*juzda:n*/ vs. StA /ħ*aqi:ba*/ “bag”).

The study of the impact of diglossia, namely the linguistic distance between SpA and StA on language and literacy development is scarce. Yet, it is receiving increasing attention, especially within the framework of *comparative linguality* and its effect on language development and metalinguistic skills in bilingual and bilectal children (Rowe and Grohmann, 2013, 2014; Grohmann and Kambanaros, 2016; Grohmann et al., 2016). With focus on literacy development, Saiegh-Haddad and colleagues (Saiegh-Haddad, 2003, 2004, 2005, 2007; Saiegh-Haddad et al., 2011; Saiegh-Haddad and Schiff, 2016; Schiff and Saiegh-Haddad, 2017) tested the impact of the linguistic distance between Spoken and Standard Arabic on the development of literacy-related skills in Standard Arabic, including phonological awareness, pseudo word decoding, and word reading. These studies showed that the development of literacy-related phonological skills in StA Arabic is impacted by the phonological distance between SpA and StA. For instance, Saiegh-Haddad (2003) compared children's phonological awareness for Spoken Arabic as against Standard Arabic phonemes and found that, even after children's production of StA phonology had normalized, children had more difficulty isolating StA than SpA phonemes. Moreover, the decoding of pseudo words encoding letters that map StA phonemes was found to challenge first graders. These effects, formalized as the *Linguistic Affiliation Constraint* (Saiegh-Haddad, 2007) or a diglossia-effect (Saiegh-Haddad, 2017) were found to persist across the early elementary grades, to surface equally strongly on production and recognition tasks (Saiegh-Haddad et al., 2011) and to show cross- dialectal external validity (Saiegh-Haddad, 2007). Research has also endorsed the role of phonological distance in letter naming (Asaad and Eviatar, 2013), as well as in reading accuracy and speed in typically developing and in disabled readers (Saiegh-Haddad and Schiff, 2016; Schiff and Saiegh-Haddad, 2017).

Research on SLI in Arabic is rather limited (however, see Abdalla and Crago, 2008; Aljenaie, 2010; Abdalla et al., 2013; Fahim, 2017; Mahfoudhi and Abdalla, 2017; Qasem and Sircar, 2017; Shaalan, 2017) and it has not yet addressed the role of diglossia in impaired language development. The current study is one step in this direction. Specifically, it examines the impact of the lexical and phonological distance between SpA and StA on phonological memory, as indexed by performance on word and non-word repetition, in SLI and TD children, and operationalized by comparing repetition of novel vs. non-novel lexical and phonological structures. The study addresses the following questions:

1. Do Arabic SLI children underperform age-matched TD children on word and non-word repetition tasks?
2. Does the lexical and phonological distance between StA and SpA impact word and non-word repetition in TD and SLI Arabic speaking children? Specifically,
  - Is word repetition in TD and SLI children affected by lexical and phonological novelty?
  - Is non-word repetition in TD and SLI children affected by phonological novelty?

Two hypotheses will be tested. The first is the *General Phonological Deficit* hypothesis according to which SLI children are predicted to underperform TD age-matched controls on all repetition tasks regardless of linguistic distance, or novelty. This hypothesis derives from earlier evidence indicating impairment in phonological processing in SLI children compared with their age-matched peers. The second hypothesis is the *Specific Linguistic Distance* hypothesis, according to which, while both SLI and TD children are predicted to find novel StA phonological and lexical units significantly harder to process than non-novel SpA structures, SLI children are expected to show particularly severe difficulty with novel linguistic structures. This prediction follows from research demonstrating that literacy-related phonological processing skills in Arabic are impacted by the *Linguistic Affiliation* of the target phonological unit (Saiegh-Haddad, 2007) with StA structures being more difficult to access than SpA structures (for a review and a model, see Saiegh-Haddad, 2017), as well as evidence suggesting that reading disabled children may be more impacted by linguistic distance than TD children (Schiff and Saiegh-Haddad, 2017). Even though earlier research in this respect has focused on literacy-related skills and has, thus, targeted phonological awareness and word-level reading tasks, it is predicted that similar patterns of effects will be observed on word and non-word repetition tasks due to shared reliance on similar underlying phonological factors. Moreover, if phonological memory for StA structures is compromised, it might be reasonable to argue that previously reported difficulties with phonological awareness and reading in StA may be attributed, at least partly, to difficulties with phonological processing in memory.

## Methods

### Participants

The sample of the study consisted of a total of one hundred children: 50 SLI (25 Senior Kindergarten, SK, 1 year before the first grade, mean age 5:09, 10 Females; 25 First Grade, mean age 6;11, 10 Females) and 50 TD (25 SK, mean age 5;10, 13 Females; 25 First Grade, mean age 6;11, 10 Females). TD children were sampled from public schools in the north school district in Israel and SLI children were sampled from the same area; SLI children were recruited from *Language Centers*, which are kindergarten and day care centers serving children diagnosed by a speech and language pathologist as having developmental language disorders; First Grade SLI children were former enrolls to *Language Centers* who attend public schools in the same area. All children had normal IQ and normal hearing levels. No child had reported developmental, neurological, or psychological problems. Data collection took place during the winter-spring of 2016. Authorization was obtained from the office of the chief scientist of the Ministry of Education. Written parental consent was obtained from all children participating in the study.

In order to confirm earlier screening and to validate the specificity of the SLI children‘s difficulties in the domain of language in comparison to the age-matched TD control group, all children were screened with ALEF (*Arabic Language: Evaluation of Function*), a language screening battery created by a US team and validated based on a normative sample of children 3–9 years of age from Saudi Arabia (Kornilov et al., 2016). Six ALEF tasks were used to screen for SLI: word articulation, expressive vocabulary, non-word repetition, non-word discrimination, sentence completion, and sentence imitation task. Rapid naming using RAN for colors and Forward Digit Span were also used for screening. ANOVA models conducted on the screening data showed that SLI children performed significantly lower than TD children on all eight tasks in both kindergarten and first grade samples. Moreover, a significant two-way interaction of grade by group was observed. In general, the interaction resulted from a larger gap between the two groups (SLI and TD) in kindergarten than in first trade. Word articulation, RAN and Digit Span only managed to discriminate between SLI and TD children in kindergarten but not in first grade. On all screening tasks, the performance of the SLI children fell below two standard deviations of the performance of the TD sample. Summary statistics and repeated measure ANOVA results for all screening tasks are summarized in Table A1.

### Experimental tasks

#### Word repetition

The study used a word repetition task that targeted two facets of the linguistic distance between SpA and StA: lexical distance and phonological distance. The impact of lexical and phonological distance was operationalized by comparing children's word repetition for four types of words: (a) *Identical* (−L−P: Lexically non-novel and Phonologically non-novel) e.g., /*ʔasad***/** “lion”; (b) *Cognate* (−L+P: Lexically non-novel, because the word is also used in SpA but Phonologically novel because it encodes one StA phoneme), e.g., /*bu:***ծ***a*/ “ice cream”; (c) *Lexically Unique* (+L−P: Lexically novel, because it is not used in SpA but Phonologically non-novel, because it does not encode any StA phoneme), i.e., /*sita:ra*/ “curtain”; and (d) *Lexically and Phonologically Unique* (+L+P: Lexically novel, because it is not used in SpA and Phonologically novel, because it encodes one StA phoneme), i.e., /*lia:m*/ “veil”. All four StA consonantal phonemes that are not used in the dialect of *Palestinian Arabic* targeted in this study were manipulated: interdental fricatives: voiced /ծ/, voiceless /*Ө*/, emphatic /ծ/, and uvular stop /q/. Words within each of the four categories varied systematically in length (1–4 syllables) in order to test the possible interaction between linguistic distance and word length on word repetition (Total *N* = 80 items, 20 items per category, five words per syllable-length condition). Note that each word, short and long, encoded just one StA phoneme. All words employed simple SpA syllabic structure (no consonantal clusters) and varied only in number of syllables. No case or mood inflections on ends of words were marked. Children were asked to repeat each word immediately after they had heard it presented by the experimenter, a native speaker of the SpA vernacular spoken by the children. One score was assigned for each accurate repetition and a zero score for inaccurate performance. Inaccurate performance included mispronouncing the target StA phoneme. *Alpha Cronbach* reliability across all tested words α = 0.96.

#### Non-word repetition

The impact of linguistic distance on non-word repetition was only addressed by targeting phonological distance. This is obviously because non-words do not have any lexical status. The effect of the phonological distance was operationalized by comparing non-word repetition for two types of words: (a) Phonologically novel (+P: encoding one StA phoneme), e.g., /*ma:*ħ*i*Θ/ and (b) Phonologically non-novel (−P, depicting only SpA phonemes), e.g., /*fanazu:n*/ (Total *N* items = 56 items, 28 items per category, 7 items per syllable-length condition). Non-words within each category varied systematically in length (1–4 syllables), so as the possible interaction of phonological distance by word length may be tested. All words employed simple SpA syllabic structure and varied only in syllabic length. Children were asked to repeat each non-word after it had been presented orally by the experimenter, a native speaker of the SpA vernacular spoken by the children. One score was assigned for accurate performance and a zero score for inaccurate performance. *Alpha Cronbach* reliability across all tested items α = 0.95.

#### Method and analytical strategy

To test our hypotheses, we used aggregate scores for the four study measurements, that is, we aggregated successful responses over the number of trials into scores per each task, and then compared children of different groups (SLI, TD) and in different grade-levels (Kindergarten, First Graders). This generated scores on a scale of zero to one hundred percent success. As this scale was within a finite range, we used the Logit transformation [i.e., log_e_(p/(1−p)), where p represents percent correct answers], which transforms 0–1 values into (−∞, +∞). To simplify the analysis, we created four groups in order to rank children's performance: SLI-Kindergarten, TD-Kindergarten, SLI-First Graders, TD-First graders. We used a repeated measure ANOVA model and a *post-hoc* ranking with the Bonferroni correction (α/4) to determine higher vs. lower performing groups (significance difference subject to *p* < 0.05). For the repeated measure we used, mainly, the word type scores: 1. identical, 2. cognate, 3. lexically unique, and 4. lexically and phonologically unique. The four repeats appeared under two variables: lexical novelty [1(3,4) vs. 2(3,4)], and phonological novelty [3(1,2) vs. 4(1,2)]. For each performance measurement, one way, two-way, and three-way repeated measure ANOVA models were performed to capture group ranking, and the interactions between group, lexical novelty and phonological novelty, if existed. Note that actual sub-group means of success rates are reported, which include ranking using the Latin letter method (“a” for the lowest rate, and so on) as superscript. Tests for main and interaction effects (*F*-tests) are reported based on the log transformed scale.

## Results

### Overall differences between SLI and TD children

The first question addressed in this study pertained to differences between kindergarten and first grade SLI and TD children in word and non-word repetition. Table 1 presents sub-group means and standard deviations for total scores as well as *post-hoc* ranking results.

**Table 1:** Summary Statistics and *post-hoc* ranking results for word and non-word repetition.

	**Kindergarten**			**First Grade**			**Both**			**Group Effect**
	**SLI-SK** **(*N* = 25)**	**TD-SK** **(*N* = 25)**	**Both** **(*N* = 50)**	**SLI-Gr.1** **(*N* = 25)**	**TD-Gr.1** **(*N* = 25)**	**Both** **(*N* = 50)**	**SLI** **(*N* = 50)**	**TD** **(*N* = 50)**	**All** **(*N* = 100)**	***F $\eta_{p}^{2}$***
Word Repetition	63.30^a^ (14.28)	88.45^b^ (9.72)	75.88 (17.54)	90.15^b^ (8.89)	97.80^c^ (3.27)	93.98 (7.67)	93.13 (8.60)	84.93 (16.25)	76.73 (17.96)	59.24^***^ 0.65
Non-word Repetition	46.36^a^ (14.27)	82.79^c^ (12.20)	64.57 (22.61)	71.21^b^ (11.85)	92.86^d^ (5.97)	82.04 (14.34)	87.82 (10.78)	73.30 (20.78)	58.79 (18.06)	52.28^***^ 0.62

^*^p < 0.05, ^**^p < 0.01,

*** *p < 0.001*.

*Superscripted letters indicate post-hoc mean ranking subject to Bonferroni correction. Standard deviations in parentheses*.

Table 1 shows all sub-group means of success rates. *Post-hoc* mean ranking as represented by Latin letters shows that, for word repetition, kindergarten SLI children achieved the lowest grades on average (a) and kindergarten TD children were the second lowest (b); first grade SLI children aligned with their kindergarten counterparts (b), whereas first grade TD children received the highest scores among all groups (c). As for non-word repetition, kindergarten SLI children always received the lowest scores, but kindergarten TD children performed better than SLI first graders (c over b). TD first graders were highest on non-word repetition (d).

### Word repetition: lexical and phonological distance effects

The second and main question addressed in this study pertained to the effect of the lexical and phonological distance between SpA and StA on repetition in Arabic diglossia. In order to address this question in the repetition of real words, a series of repeated measure ANOVA models were conducted on items within each syllable-length condition separately; These analyses compared, in addition to the four groups, the two sets of lexical and phonological categories, and two-way and three-way interaction effects across the categorical sets. Table 2 provides summary statistics and by group ranking. Table 3 provides the ANOVA model main and interaction effects on the word repetition scores.

**Table 2:** Means and standard deviations of word repetition by group, word type, and syllabic length.

	**Kindergarten**			**1st grade**			**Both**		
	**SLI-SK** **(*N* = 25)**	**TD-SK** **(*N* = 25)**	**Both** **(*N* = 50)**	**SLI-Gr.1** **(*N* = 25)**	**TD-Gr.1** **(*N* = 25)**	**Both** **(*N* = 50)**	**SLI** **(*N* = 50)**	**TD** **(*N* = 50)**	**All** **(*N* = 100)**
**1 SYLLABLE**									
Identical words (−L−P)	98.40 (8.00)	100 (0.00)	99.20 (5.66)	100 (0.00)	100 (0.00)	100.00 (0.00)	100.00 (0.00)	99.20 (5.66)	99.60 (4.00)
Cognate words (−L+P)	60.80 (23.44)	87.20 (16.21)	74.00 (23.99)	88.00 (17.32)	88.40 (8.00)	93.20 (14.35)	92.80 (13.86)	74.40 (24.59)	83.60 (21.91)
Lexically Unique (+L−P)	96.80 (7.48)	99.20 (4.00)	98.00 (6.06)	100 (0.00)	100 (0.00)	100.00 (0.00)	99.60 (2.83)	98.40 (5.48)	99.00 (4.38)
Lexically and Phonologically Unique (+L+P)	57.60 (24.03)	80.00 (19.15)	68.80 (24.30)	86.40 (17.05)	96.00 (10.00)	99.00 (4.38)	88.00 (17.14)	72.00 (25.23)	80.00 (22.92)
Total	78.40^a^ (11.15)	91.60^b^ (7.32)	85.00 (11.47)	93.60^b^ (6.70)	98.60^c^ (3.69)	96.10 (5.92)	95.10 (6.74)	86.00 (11.91)	90.55 (10.66)
**2 SYLLABLES**									
Identical words (−L−P)	97.60 (6.63)	100 (0.00)	98.80 (4.80)	100 (0.00)	100 (0.00)	100.00 (0.00)	100.00 (0.00)	98.80 (4.80)	99.40 (3.43)
Cognate words (−L+P)	31.20 (26.51)	80.00 (28.87)	55.60 (36.88)	84.00 (23.09)	98.40 (5.54)	91.20 (18.14)	89.20 (22.57)	57.60 (36.28)	73.40 (34.00)
Lexically Unique (+L−P)	96.80 (7.48)	100 (0.00)	98.40 (5.48)	100 (0.00)	99.20 (4.00)	99.60 (2.83)	99.60 (2.82)	98.40 (5.48)	99.00 (4.38)
Lexically and Phonologically Unique (+L+P)	43.20 (28.10)	87.20 (18.15)	65.20 (32.28)	84.80 (17.59)	98.40 (8.00)	91.60 (15.17)	98.40 (5.48)	64.00 (31.30)	78.40 (28.38)
Total	67.20^a^ (14.00)	91.80^b^ (11.17)	79.50 (17.65)	92.20^b^ (9.47)	99.00^c^ (3.23)	95.60 (7.80)	95.40 (8.91)	79.70 (17.30)	87.55 (15.80)
**3 SYLLABLES**									
Identical words (−L−P)	96.00 (10.00)	99.20 (4.00)	97.60 (7.71)	99.20 (4.00)	100 (0.00)	99.60 (2.82)	99.60 (2.83)	97.60 (7.71)	98.60 (63.40)
Cognate words (−L+P)	29.60 (24.58)	64.80 (26.63)	47.20 (30.97)	70.40 (25.90)	88.80 (15.36)	79.60 (23.03)	76.80 (24.70)	50.00 (32.39)	63.40 (31.66)
Lexically Unique (+L−P)	92.80 (11.37)	99.20 (4.00)	96.00 (9.04)	97.60 (6.63)	99.20 (4.00)	98.40 (5.48)	99.20 (3.96)	95.20 (9.53)	97.20 (7.53)
Lexically and Phonologically Unique (+L+P)	28.80 (31.13)	70.40 (27.15)	49.60 (35.74)	76.80 (27.50)	93.60 (13.81)	85.20 (23.14)	82.00 (24.33)	52.80 (37.85)	67.40 (34.89)
Total	61.80^a^ (15.47)	83.40^b^ (12.22)	72.60 (17.59)	86.00^b^ (11.99)	95.40^c^ (6.91)	90.70 (10.78)	89.40 (11.55)	73.90 (18.36)	81.65 (17.13)
**4 SYLLABLES**									
Identical words (−L−P)	63.20 (30.38)	99.20 (4.00)	81.20 (28.11)	98.40 (5.54)	100 (0.00)	99.20 (3.96)	99.60 (2.83)	80.80 (27.98)	90.20 (21.93)
Cognate words (−L+P)	32.00 (24.49)	74.40 (24.17)	53.20 (32.23)	81.60 (21.54)	95.20 (8.72)	88.40 (17.65)	84.80 (20.82)	56.80 (33.89)	70.80 (31.32)
Lexically Unique (+L−P)	56.80 (28.68)	93.60 (12.54)	75.20 (28.73)	93.60 (11.14)	100 (0.00)	98.80 (8.44)	96.80 (9.35)	75.20 (28.45)	86.00 (23.70)
Lexically and Phonologically Unique (+L+P)	31.20 (25.22)	80.80 (24.14)	56.00 (34.99)	81.60 (23.04)	97.60 (6.63)	89.60 (18.62)	89.20 (19.47)	56.40 (34.92)	72.80 (32.60)
Total	45.80^a^ (23.88)	87.00^b^ (13.23)	66.40 (28.25)	88.80^b^ (11.30)	98.20^c^ (3.19)	93.50 (9.49)	92.60 (11.08)	67.30 (28.52)	79.95 (25.00)

*+L, Lexically Novel; −L, Lexically non-novel; +P, Phonologically Novel; −P, Phonologically Non-novel; SK, Senior Kindergarten. Superscripted letters indicate post-hoc mean ranking subject to Bonferroni correction. Standard deviations in parentheses*.

**Table 3:** Repeated measure ANOVA model results of Word Repetition by each word length set separately: Lexical and Phonological Novelty are used as within subject factors.

	**Df**	**1 syllable**		**2 syllables**		**3 syllables**		**4 syllables**	
		***F***	**$\eta_{p}^{2}$**	***F***	**$\eta_{p}^{2}$**	***F***	**$\eta_{p}^{2}$**	***F***	**$\eta_{p}^{2}$**
Group	3.96	35.89^***^	0.53	39.59^***^	0.55	31.22^***^	0.49	65.94^***^	0.67
Lexical Novelty	1.96	9.54^**^	0.09	2.21	0.02	1.47	0.23	0.34	0.004
Lexical Novelty X Group	3.96	0.76	0.02	1.63	0.05	1.86	0.14	0.86	0.03
Phonological Novelty	1.96	154.18^***^	0.62	155.88^***^	0.62	265.12^***^	0.73	78.63^***^	0.45
Phonological Novelty X Group	3.96	20.34^***^	0.39	31.27^***^	0.49	19.01^***^	0.37	3.20^*^	0.09
Lexical Novelty X Phonological Novelty	1.96	2.31	0.02	6.26^*^	0.06	12.00^**^	0.11	17.28^***^	0.15
Lexical Novelty X Phonological Novelty X Group	3.96	0.51	0.02	2.82^*^	0.08	0.50	0.02	1.13	0.034

* *p < 0.05*,

** *p < 0.01*,

*** p < 0.001;

*Repeated, word novelty, phonological novelty. Group (SLI-SK, TD-SK, SLI-Gr1, TD-Gr1) is used as a between subject factor*.

**Figure 1:**
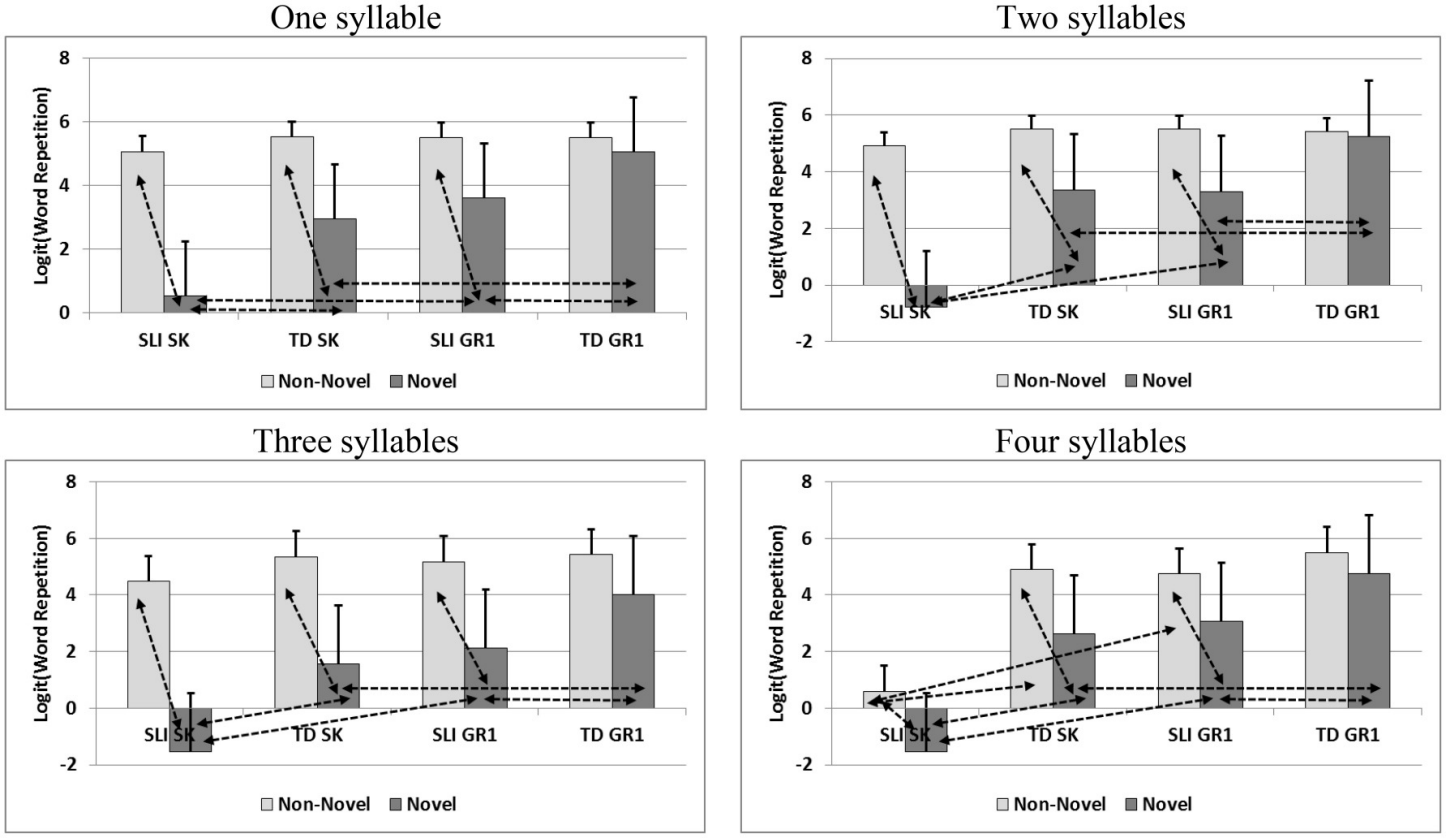
Interaction analyses of word repetitions scores (Log transformed) by syllable length. Positive standard deviation in error bars; Double head arrows for a significant difference at *p* < 0.05.

Table 2 shows a consistent pattern of ranking across syllable length sets. Younger SLI children in kindergarten yielded the lowest scores (a); older SLI children at first grade performed similarly to younger TD children at kindergarten (b), and older TD children at first grade performed the highest in word repetition (c). Beyond the group main effect across all syllable lengths (1–4 syllables), Table 3 shows that lexical novelty had a significant effect on word repetition only for shorter (1 syllable) words (*F* = 9.54, *p* < 0.01). In contrast with lexical novelty, phonological novelty had a consistent effect on word repetition across all syllable-length conditions (1 syllable: *F* = 154.18, *p* < 0.001; 2 syllables: *F* = 115.88, *p* < 0.001; 3 syllables: *F* = 265.12, *p* < 0.001; 4 syllables: *F* = 78.63, *p* < 0.001). Moreover, the two-way interaction of phonological novelty by group was significant across all syllable-length conditions as well (1 syllable: *F* = 20.34, *p* < 0.001; 2 syllables: *F* = 31.27, *p* < 0.001; 3 syllables: *F* = 19.01, *p* < 0.001; 4 syllables: *F* = 3.20, *p* < 0.05). The interaction between lexical and phonological novelty was found significant in two, three, and four syllable words. As the focus of this study is on the group main and interactive effect with novelty, we did not proceed with decomposing the latter interaction. Moreover, interactions that do not involve the group effect might suggest that performance differences were due to a hidden group effect. We present the sources of the former interactions in Figure 1. Figure 1 presents the major sources of these interaction effects. In this figure and the following figures for decomposing interactions, the double head arrows represent two significantly different sub-group means, where each head marks one sub-group mean. The usual *p* < 0.05 criterion was used to show group differences. Figure 1 shows that across syllable lengths, SLI children at both kindergarten and first grade, as well as kindergarten TD children performed differently when items were phonologically novel vs. non-novel. The sources of these interactions became stronger as number of syllables increased. That is, differences between performance of repeating phonologically novel vs. non-novel words were clear across the groups, but when words were of four syllables, a difference in words with non-novel phonemes was also found between SLI-SK, on the one hand, and both TD-SK and SLI-GR1. Lastly, we decomposed the sources of the three-way interaction between group, lexical novelty, and phonological novelty. We found that except for a major success rate reduction in word repetition among kindergarten SLI children when novel and non-novel phonemes in lexically non-novel words, the other potential sources were similar.

### Non-word repetition: phonological distance effects

Performance on the non-word repetition task was analyzed using the repeated measure ANOVA model on each syllable length condition separately. Table 4 shows descriptive statistics separated by syllable length and sub-group mean ranking. Regardless of syllable length, SLI children at kindergarten performed consistently lower in comparison to others (a) First grade SLI children performed higher than their kindergarten SLI counterparts (b) across all syllable lengths, but as syllable length increased (2–4 syllables), kindergarten TD children performed more successfully than both SLI groups (c); first grade TD children were the most successful in non-word repetition across all syllable lengths (d). Table 5 presents the ANOVA model results.

**Table 4:** Means and Standard Deviations for Non-word Repetition by group, phonological novelty, and syllable length.

	**Kindergarten**			**1**^**st**^ **grade**			**Both**		
	**SLI-SK** **(*N* = 25)**	**TD-SK** **(*N* = 25)**	**Both** **(*N* = 50)**	**SLI-Gr.1** **(*N* = 25)**	**TD-Gr.1** **(*N* = 25)**	**Both** **(*N* = 50)**	**SLI** **(*N* = 50)**	**TD** **(*N* = 50)**	**All** **(*N* = 100)**
**1 SYLLABLE**									
Phonologically non-novel −P	94.29 (10.10)	98.29 (4.74)	96.29 (8.07)	96 (7.74)	100 (0.00)	98.00 (5.78)	99.15 (3.43)	95.14 (8.95)	97.14 (7.03)
Phonologically novel +P	39.43 (23.79)	85.14 (21.22)	62.29 (32.11)	81.14 (23.59)	99.43 (2.86)	90.29 (19.02)	92.29 (16.63)	60.29 (31.52)	76.29 (29.79)
Total	66.86^a^ (13.66)	91.71^b^ (11.79)	79.29 (17.81)	88.57^b^ (12.71)	99.71^c^ (1.43)	94.14 (10.57)	95.71 (9.24)	77.71 (71.57)	86.71 (16.37)
**2 SYLLABLES**									
Phonologically non-novel −P	89.71 (13.98)	98.86 (3.96)	94.29 (11.18)	93.14 (8.37)	100 (0.00)	96.57 (6.81)	99.43 (2.83)	91.43 (11.55)	95.43 (9.28)
Phonologically novel +P	31.43 (20.62)	78.29 (23.73)	54.86 (32.32)	72.00 (24.22)	99.43 (2.86)	85.71 (21.98)	88.86 (19.85)	51.71 (30.26)	70.29 (31.57)
Total	60.57^a^ (14.30)	88.57^c^ (11.48)	75.57 (19.10)	82.57^b^ (12.39)	99.71^d^ (1.43)	91.14 (12.29)	94.14 (9.86)	71.57 (17.29)	82.86 (18.02)
**3 SYLLABLES**									
Phonologically non-novel −P	57.14 (27.97)	96 (7.74)	76.57 (28.24)	82.29 (11.87)	98.29 (6.28)	90.29 (12.39)	97.14 (7.07)	69.71 (24.77)	83.43 (22.77)
Phonologically novel +P	20.00 (19.78)	68.00 (26.17)	44.00 (33.39)	45.14 (24.98)	85.71 (15.97)	65.43 (29.16)	76.86 (23.25)	32.57 (25.66)	54.71 (32.99)
Total	38.57^a^ (21.03)	82.00^c^ (12.90)	60.29 (27.91)	63.71^b^ (14.57)	92.00^d^ (9.29)	77.86 (18.72)	87.00 (12.22)	51.14 (21.95)	69.07 (25.24)
**4 SYLLABLES**									
Phonologically non-novel −P	30.86 (27.87)	84.57 (16.96)	57.71 (35.46)	65.71 (16.50)	82.29 (19.02)	74.00 (19.51)	83.43 (17.88)	48.29 (28.70)	65.86 (29.63)
Phonologically novel +P	8.00 (13.09)	53.14 (27.51)	30.57 (31.22)	34.29 (25.75)	77.71 (17.54)	56.00 (30.93)	65.43 (25.99)	21.14 (24.19)	43.29 (33.45)
Total	19.43^a^ (18.27)	68.86^c^ (19.55)	44.14 (31.20)	50.00^b^ (17.98)	80.00^c^ (16.62)	65.00 (22.87)	74.43 (18.82)	34.71 (23.67)	54.57 (29.17)

*Superscripted letters indicate post-hoc mean ranking subject to Bonferroni correction. Standard deviations in parentheses*.

**Table 5:** Repeated measure ANOVA model results for Non-word Repetition for each syllable-length condition separately.

	**df**	**1 syllable**		**2 syllables**		**3 syllables**		**4 syllables**	
		***F***	**$\eta_{p}^{2}$**	***F***	**$\eta_{p}^{2}$**	***F***	**$\eta_{p}^{2}$**	***F***	**$\eta_{p}^{2}$**
Group	3.96	28.52^***^	0.47	46.90^***^	0.59	62.11^***^	0.66	45.02^***^	0.59
Phonological Novelty	1.96	87.10^***^	0.00	81.62^***^	0.46	98.88^***^	0.51	82.21^***^	0.46
Phonological Novelty X Group	3.96	19.69^***^	0.00	12.63^***^	0.28	0.88	0.03	2.44	0.07

^*^p < 0.05, ^**^p < 0.01,

*** *p < 0.001; Repeated, phonological novelty*.

**Figure 2:**
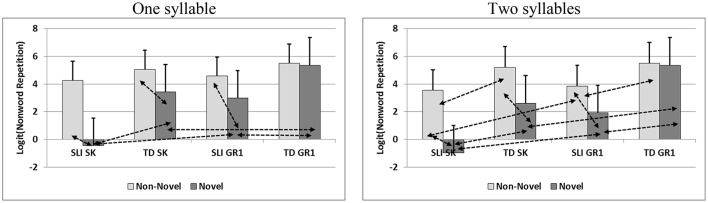
Interaction analyses of nonword repetitions scores (Log transformed) by syllable length. Positive standard deviation in error bars; Double head arrows for a significant difference at *p* < 0.05.

Results show a large and consistent group difference on all syllable-length conditions (1 syllable: *F* = 28.52, *p* < 0.001; 2 syllables: *F* = 46.90, *p* < 0.001; 3 syllables: *F* = 62.11, *p* < 0.001; 4 syllables: *F* = 45.02, *p* < 0.001). Phonological novelty main effect was found significant across all syllable lengths as well (1 syllable: *F* = 87.10, *p* < 0.001; 2 syllables: *F* = 81.62, *p* < 0.001; 3 syllables: *F* = 98.88, *p* < 0.001; 4 syllables: *F* = 82.21, *p* < 0.001). The interaction of group by phonological novelty was significant only when non-words were short (1 syllable: *F* = 19.69, *p* < 0.001; 2 syllables: *F* = 12.63, *p* < 0.001). Figure 2 presents the sources of these interactions. Differences between non-words with non-novel and novel phonemes were found in the first three sub-groups: SLI-SK, SLI-Gr1, TD-SK, as in the word repetition analysis. Moreover, 1-syllable and 2-syllable non-words with novel phonemes yielded significantly lower scores in the SLI group than in the TD group at both kindergarten and first grade. The same pattern was observed in 2-syllable non-novel non-words. As for three and four syllable non-words, no interactions in non-word repetition were found.

## Discussion

The current study is an investigation of the impact of diglossia on phonological memory in Arabic speaking SLI children and in TD age-matched controls. Specifically, it examines the impact of the lexical and phonological distance between SpA and StA on phonological memory, indexed as performance on word and non-word repetition, and operationalized as a comparison between items depicting novel vs. non-novel lexical and phonological structures. The current study defines novelty in functional sociolinguistic terms, rather than in absolute structural terms, as availability of a certain linguistic unit in the standard/written language but not in everyday spoken speech. In turn, according to this definition, non-novel units are expected to be associated with more active practice in speaking and more exposure and entrenchment. This sociolinguistically-defined phonological property, is characteristic of the linguistic reality of children raised in Arabic diglossia, and is probably also applicable to children in many other bilectal and bilingual settings. The aim of the current study is to test the role of novelty, as defined above, on developmental language impairment in Arabic.

### General phonological memory deficits in Arabic SLI

The current study set out to investigate phonological deficits in Arabic SLI with particular focus on the impact of linguistic distance. One hypothesis that the current study tested was the *General Phonological Deficit* hypothesis according to which SLI children are expected to underperform TD children on all tasks requiring phonological processing in memory: word repetition and non-word repetition, especially for long words, and regardless of linguistic distance, or novelty. This hypothesis derives from research indicating that, when compared with their age-matched controls, SLI children show clear phonological deficits, including deficits in auditory phonological processing and memory (Gathercole and Baddeley, 1990; Tallal et al., 1991, 1993; Joanisse and Seidenberg, 1998; Newbury et al., 2005), phonological representations and awareness (Thatcher, 2010; Claessen and Leitão, 2012), phonological decoding (Tambyraja et al., 2015), and phonological learning. This was demonstrated in English monolinguals, as well as in monolingual speakers of several other languages (e.g., Newbury et al., 2005; de Bree et al., 2007; Dispaldro et al., 2013). Phonological deficits were also reported in the two languages of bilingual SLI children (e.g., Gutiérrez-Clellen and Simon-Cereijido, 2010; Windsor et al., 2010).

In line with the above, the results of the current study focusing on word and non-word repetition show that Arabic speaking SLI children, who are raised in a diglossic (bilectal) setting, fare significantly lower than their age-matched controls on both tasks implicating phonological processing in memory: word repetition and non-word repetition, and even when the phonological forms targeted are limited to SpA. These results accord with the *General Phonological Deficit hypothesis* and extend earlier findings in demonstrating that Arabic speaking SLI, like monolingual SLI children raised with just one language or language variety, show a deficit in phonological memory. These findings align with theories of processing deficits which posit that SLI may be grounded in a deficit in input processing capacity (Gathercole and Baddeley, 1990; Tallal et al., 1991, 1993; Joanisse and Seidenberg, 1998).

Whereas phonological deficits were observed in the SLI group at both kindergarten and first grade, the results showed that, in both SLI and TD groups, the performance of first grade children was higher than the performance of their kindergarten peers. Moreover, while SLI first graders aligned with TD kindergarteners in word repetition, non-word repetition of SLI children at first grade was lower than the performance of TD children at kindergarten. Altogether, these findings indicate a positive impact of first grade exposure to StA and to the shallow vowelized Arabic orthography on phonological processing in Arabic in both SLI and TD children, yet a weaker effect for SLI children. It is noteworthy that evidence for the impact of literacy on phonological processing among English speaking SLI children is not clear. For instance, Thatcher (2010) found no gains in phonological awareness in SLI children, as opposed to TD age-matched peers, between kindergarten and first grade. This finding, however, has to be interpreted within the context of literacy instruction in English. Literacy instruction in English speaking children (at least in the US) starts in kindergarten, and this might reduce the extent of gain observed between kindergarten and first grade. In the Arabic context in Israel, very little exposure to StA and instruction in literacy in StA takes place in kindergarten. Rather, StA language and literacy instruction starts mainly in the first grade. This difference, together with the phonological disparity between Spoken Arabic and Standard Arabic, including in the consonantal system which was targeted in the current study, might be responsible for the different patterning of results. In other words, because some of the Standard Arabic phonemes are absent from the phonological system of Spoken Arabic, exposure to literacy in the first grade might help children represent these phonemes more accurately, especially as learning to read entails learning the different letters that map these phonemes. Thus, the specific gain in phonological processing observed in the first grade in our sample might reflect the combined effect of two factors: (a) intensive exposure to StA in the first grade in the light of diglossia and (b) experience with the shallow orthography of vowelized Arabic which maps Standard Arabic phonology. These factors might impact on both input and output phonological skills, including quality of phonological representations, efficiency of phonemic encoding, phonemic segmentation and blending, as well as articulatory motor planning (Hassunah et al., 2017; Saiegh-Haddad, 2017). Indeed, earlier research on Arabic reports marked first grade gains in phonological awareness for production tasks, such as phoneme isolation among TD children (Saiegh-Haddad, 2003). Future research is warranted that tests the contribution of first grade exposure to StA and of experience with vowelized Arabic on input and output phonological skills among TD vs. SLI children, especially in light of the phonological disparity in Arabic between SpA, the language of everyday speech and StA, the language encoded in print. This research should try to address the problem of ceiling levels of performance, especially among TD kindergarten children, which surfaced in the current study and which limit the validity and generalizability of conclusion regarding impact of literacy on phonological memory in SLI vs. TD children.

### Diglossia reflexes on word and non-word repetition of novel phonological and lexical forms in Arabic

The results discussed in the previous section are based on overall scores and do not take into account possible differences in phonological memory that may be associated with linguistic distance, namely availability or not of the linguistic unit in the spoken variety used by children in everyday speech. This question is receiving increasing attention especially within the framework of *comparative linguality* and effects on language development and metalinguistic skills in bilingual and bilectal children (Rowe and Grohmann, 2013, 2014; Grohmann and Kambanaros, 2016; Grohmann et al., 2016). Arabic diglossia offers a natural setting for testing this question in developmental language impairment. The current study focuses on phonological and lexical distance and on its effect on phonological processing in memory. Phonological memory is tested using word and non-word repetition, and the impact of phonological and lexical distance is operationalized by comparing memory for novel StA phonological and lexical structures that are not within the spoken repertoire of children with non-novel SpA units.

To address the impact of lexical and phonological distance on real word repetition four types of words were compared: (a) *Identical* (−L−P: Lexically non-novel and Phonologically non-novel); (b) *Cognate* (−L+P: Lexically non-novel but Phonologically novel); (c) *Lexically Unique* (+L−P: Lexically novel but Phonologically non-novel); and (d) *Lexically and Phonologically Unique* (+L+P: Lexically novel and Phonologically novel). Moreover, because phonological memory is sensitive to the effect of word length (Gathercole and Baddeley, 1990), an attempt was made to dissociate the effect of this factor from the effect of novelty by manipulating phonological and lexical distance within each of four syllable-length conditions (1–4 syllable long items) independently. The results obtained from the manipulation of phonological and lexical novelty on real word repetition reveal a significant effect of lexical novelty on word repetition for short words (1-syllable long), and no interaction with group. The lexical novelty effect was reflected in the finding that word repetition of Identical and Cognate words was more accurate than the repetition of Unique words, both phonologically novel and phonologically non-novel. This effect, however, was limited to short one-syllable words and did not extend to longer words. This means that when the word was lexically not novel and short, it was easier for children to repeat than a lexically novel word, and regardless of phonological novelty. This finding mimics the word frequency/familiarity effect observed in the literature (Garlock et al., 2001; Gathercole, 2006); Identical and Cognate words are used in SpA and are thus familiar to children, whereas lexically novel words which are only used in StA are naturally less familiar, and it implies reliance on lexical feedback to aid storage and processing in memory for lexically non-novel short words. However, when the word was both longer than one syllable and lexically novel a bottleneck effect was observed and lexical feedback could no longer avert memory decay. Interestingly, the results did not show these patterns to be unique for SLI children, or even more prominent in SLI than in TD children, probably indicating general, rather than SLI-specific patterns of quantitative memory span and qualitative lexical distance effects.

Unlike lexical distance, large, and consistent effects were observed for phonological distance on word repetition among SLI children at both kindergarten and first grade, as well as among TD kindergarten children. This effect was evident across all syllable-length conditions: short and long, and it interacted group. Non-word repetition showed a similar effect of phonological distance in the same groups of children and across all syllable-length sets, yet a significant interaction with group for only 1-syllable and 2-syllable non-words. In other words, across syllable-length sets, SLI children at both kindergarten and first grade performed more poorly when the word encoded a StA phoneme than when it only encoded SpA phonemes. This was not the case among TD children who only showed this pattern in kindergarten but not in first grade. These results imply a strong role of phonological distance in impeding phonological memory in children in general, but also a stronger effect among SLI than TD children. These findings are in harmony with earlier reports of the effect of phonological distance on literacy-related phonological skills, including phonological awareness (Saiegh-Haddad, 2003, 2004, 2007; Saiegh-Haddad et al., 2011), phonological naming (Asaad and Eviatar, 2013), phonological recoding of pseudo words (Saiegh-Haddad, 2005), as well as word decoding accuracy and speed in typically developing children (Saiegh-Haddad and Schiff, 2016) and in developmental dyslexia (Schiff and Saiegh-Haddad, 2017). More importantly, the results accord with the *Linguistic Distance Hypothesis* stipulating a stronger impact of linguistic distance on SLI children's phonological memory skills. Given the observed patterns of results according to which lexical distance was found to show a limited effect on the repetition of short word only, and no interaction with group (SLI vs. TD), it might be more appropriate to refer to a *Specific Phonological Distance* hypothesis rather than a *Specific Linguistic Distance* Hypothesis. Future research should test the role of other aspects of phonology and lexicon on phonological processing in SLI in order to corroborate this hypothesis.

It is noteworthy that the results of the current study reveal a significant difference between memory for phonologically novel and non-novel words in SLI children in kindergarten as well as in first grade, yet only in kindergarten among TD children but not in the first grade. Moreover, while the repetition of phonologically novel words and non-words improved significantly between kindergarten and first grade among TD children yielding a non-significant difference between the two types of stimuli, the difference between the two sets of words remained significant in the SLI first grade children. This finding implies yet again a weaker effect of exposure to StA and to literacy in Arabic on SLI than on TD children's general phonological memory, and on memory for novel StA phonological units in particular. We reiterate that this interpretation must be treated with great caution given ceiling levels of performance, especially among TD kindergarteners, and specifically when words were non-novel.

Besides the observed effect of phonological and lexical distance, the results of the current study reveal different patterns of interactions of these factors with group in stimuli that vary in syllabic length. These patterns imply that linguistic distance and word length might constitute two different processing constraints on phonological processing in Arabic. The results showed that in the case of non-novel items, the repetition of 1–3 syllable words and 1-syllable non-words yielded similar repetition accuracy scores in SLI and in TD children, in kindergarten and in first grade, failing hence to discriminate between the two groups; non-novel words managed to tease the groups apart at kindergarten only when they were 4-syllables long, and non-words managed to do so at both kindergarten and first grade when they were 2-syllables long. These results are not commensurate with those reported among English speaking children where monosyllabic non-words (which are comparable to the non-novel non-words used in our study; they did not encode any novel phoneme but only depicted a novel composition of phonemes) were found to yield significant differences between SLI and TD children (Gathercole and Baddeley, 1990). Differences between the patterns observed in the current study and those reported for English speaking children might be attributed to differences between the two languages in phonological complexity with English monosyllabic words depicting complex clustered onsets and codas, in contrast with Arabic where such clusters are limited and were thus excluded (Gibson et al., 2015). At the same time, Gathercole and Baddeley (1990) report that while the word repetition deficit among SLI children was observed in monosyllabic words, it became more robust when longer 3–4 syllable words were used (Dollaghan and Campbell, 1998). This observation is corroborated by our findings which reveal that phonologically non-novel Arabic words became sensitive to language impairment, in kindergarten and first grade children, only when they were 4-syllables long; Shorter phonologically non-novel words failed to tease apart an SLI from a TD child in both grades. It is interesting to note that Arabic is a multi-syllabic language, it is a consonantal root-pattern based language with most content words and even some function words made up through the indigitation of consonantal roots within mutlisyllabic prosodic templates. An analysis of the phonological structure of Spoken Arabic revealed that only 16.5% of the words in the Spoken Arabic lexicon of 5-year-old children were monosyllabic words, whereas 61.1% were bi-syllabic words, 21.3% were tri-syllabic (Saiegh-Haddad and Spolsky, 2014). Moreover, the multisyllabic lexicon of Arabic is organized and constrained by highly regular morpho-phonological templates—derivational and inflectional word-patterns functioning as fixed phonological/prosodic word envelopes and capturing the syllabic structure of the word (Saiegh-Haddad and Geva, 2008; Saiegh-Haddad and Henkin-Roitfarb, 2014). This morpho-phonological property has been shown to result in word patterns being accessed and employed rather early in linguistic processing amongst Arabic speaking children (Saiegh-Haddad, 2013, 2017; Taha and Saiegh-Haddad, 2016, 2017; Saiegh-Haddad and Taha, 2017). Given this, it would be reasonable to expect Arabic speaking children, both SLI and TD, to show relative ease in processing longer words, and regardless of linguistic distance. This conclusion is in accordance with earlier research showing that speakers of a multi-syllabic language, like Spanish, find it easier to process long strings of verbal input in both Spanish and English than those coming from a linguistic background that does not feature as many multi-syllabic words (Gibson et al., 2015).

Phonologically novel words and non-words were found to behave genuinely differently from non-novel words and to show sensitivity to language impairment even in the case of short words. In the case of both words and non-words, the results show that repetition of even the shortest 1-syllable novel word yielded a significant difference between SLI and age-matched TD controls, both in kindergarten and in the first grade. These results highlight the role of phonological distance, as it is defined in this study, as an important linguistic constraint on phonological processing in diglossic Arabic, and as a phonological complexity factor that is particularly sensitive to language impairment in this language setting. These results also imply that the quantitative word length factor and the qualitative phonological distance factor might constitute two independent constraints on phonological memory. This has important theoretical and clinical implications. To name just a few, the results imply that diagnosis of SLI should treat length and phonological distance separately. Short words encoding a novel phoneme prove successful in discriminating between SLI and TD children at both kindergarten and first grade. However, it is only when the word is very long (4 syllables long) that a phonologically non-novel real word can dissociate the two groups, and only at kindergarten. In the same way, a non-novel non-word need be 2-sylalbles long to manage to tease the groups apart. Moreover, these constraints should be manipulated in different ways to diagnose groups in kindergarten vs. first grade. Note that phonologically non-novel 4-syllable words failed to tease SLI and TD children apart at first grade but they managed to do so in kindergarten.

Children with specific language impairment are particularly sensitive to phonological complexity in their language and their performance drops when complexity increases (dos Santos and Ferré, 2016). The current results demonstrate a particular complexity that Arabic SLI children are confronted with. This is phonological distance which was found to have an overarching effect on the repetition of all words: real words and non-words, short and long. This finding is remarkable because linguistic distance parameters are usually not heeded when phonological complexity is defined and when measures of phonological representation, processing, or awareness are used with bilectal or bilingual children (Russak and Saiegh-Haddad, 2011, 2017). Research has shown that linguistic factors impact phonological processing skills in typically developing and in SLI children (Munson et al., 2005; Graf Estes et al., 2007). SLI children were even found to be more sensitive than typically developing children to linguistic manipulations within tasks (Munson et al., 2005). This is probably due to the genuinely linguistic nature of their deficit, and due to the effect of the quality of the long-term store of linguistic structures on phonological processing (Newbury et al., 2005; Zourou et al., 2010). The current study showed that lexical distance is another important factor that has an impact on the repetition of words, short real words in particular, among bilectal children. These effects should, therefore, become an indispensable part of the characterization of the repetition deficit in SLI and in specifying its underlying cognitive and linguistic basis.

Two issues are in order. First, the linguistic distance factor that the current study has targeted may be genuinely different from the familiarity/novelty factor often manipulated when wordlikeness is tested. This is because linguistic distance does not imply absolute lack of familiarity with a given linguistic unit. Rather, gradable levels associated with degree of language experience and practice, as well as quantity and quality of spheres of use of the two language varieties. This is a sociolinguistic variable that characterizes the linguistic reality of language development in diglossia (Saiegh-Haddad, 2012). Second, with respect to phonological distance, even when an analogy with wordlikeness is held up, phonological distance was operationalized differently in the current study and it referred to whether the phonological form of the word encoded a novel StA phoneme, rather than whether the compositional structure of the word was novel. This aspect of novelty has not been tested before, and the results of the current study show that it has a strong and persistent effect on phonological processing in children and especially so in SLI children. This finding has clear theoretical implications, as well as important practical implications.

Theoretically, the results imply that theories of language development and impairment cannot be agnostic to the sociolinguistic context within which language acquisition is embedded and to the distributional nature of linguistic knowledge and representation that is true of bilingual and bilectal children. Moreover, the findings imply that cognitive deficits, such as memory and metalinguistic skills are not purely cognitive or insensitive to language-specific linguistic factors. Rather, they are impacted by linguistic representations, and in as much as these linguistic representations are inaccurate or unstable any operation on or access to these representations should be expected to be more difficult to demonstrate (Swan and Goswami, 1997; Foy and Mann, 2001).

In terms of practical and clinical implications, the results demonstrate that the phonological deficits observed in SLI are exacerbated in the Arabic context by linguistic distance making phonological processing particularly challenging for Arabic speaking children. In turn, early intervention with Arabic speaking SLI children should probably suspend attention to these units and should begin, instead, with those phonological and lexical units that are familiar to children from their spoken Arabic vernacular. At the same time, after some basis of phonological representations and processing has been established, particular focus to the phonological distance between SpA and StA should be given particular attention, especially when children start learning to read and given the fact that literacy acquisition in Arabic happens only in the standard variety (Saiegh-Haddad and Everatt, 2017).

Another practical implication concerns diagnosis of and intervention with SLI. The results of the study indicate that novel phonological units are particularly difficult for SLI children and in kindergarten in particular, and this effect surfaces even when short words are employed. For instance, the results of the non-word repetition task showed that one syllable non-novel non-words yielded similar repetition scores in all four groups tested, whereas the repetition of two syllable non-novel non-words and 1–2 syllable novel non-words, yielded different scores in the four groups. All this implies that to diagnose young children with SLI, attention to novel phonological units in conjunction with word length is warranted, and it should be given thorough attention in task construction and performance interpretation, especially as the two factors may be used to make different inferences regarding the nature of the underlying difficulty and hence different implications for intervention. Word length is a quantitative constraint on memory capacity and an effect of length in the absence of a phonological distance effect might imply difficulty with memory span. In contrast, phonological distance effect even in the case of short words might imply phonological representational quality problems disrupting storage and processing in memory. The effect of this factor is naturally exacerbated in longer words as our results show.

## Conclusion and limitations

The results of the current study show that two factors that pertain to Arabic diglossia affect phonological storage in working memory in Arabic speaking TD and SLI children. These are lexical distance and phonological distance. Moreover, the impact of these factors on phonological memory surfaces in different ways in shorter and longer words implying, hence, an interaction between the quantitative length memory span factor and the qualitative linguistic distance representational factor.

It is to be remembered that the evidence we report in this article is based on a small sample size and on a cross-sectional design. These are two critical limitations on the generalizability of the results we report. Moreover, the results of the current study are based on Arabic native speaking children living in Israel, and they should be replicated among speakers of Arabic in other regions in the Arabic-speaking world. Finally, phonological and lexical distance was operationalized in this study based on a local dialect of *Palestinian Arabic* vernacular spoken in the north of Israel; Linguistic distance is a variable concept and it is realized differently in different regions and with different spoken Arabic vernaculars. Future research that replicates the design of the current study but targets other phonological and lexical structures is warranted in order to demonstrate the external validity of the results reported in this study. Finally, despite the fact that our SLI sample was screened based on various language tasks, including phonology and lexicon, many more of these tasks tapped into phonological processing. Thus, the possibility that our SLI sample had more phonological deficits than other language deficits cannot be precluded.

## Author contributions

This paper was co-authored by ES-H and OG-D and is based on a doctoral dissertation conducted by the second author under the supervision of the first author.

### Conflict of interest statement

The authors declare that the research was conducted in the absence of any commercial or financial relationships that could be construed as a potential conflict of interest.

## Footnotes

^1^As argued by some (e.g., Snowling and Hulme, 1989; Snowling et al., 1991), quantitative phonological memory limitations may not be the only phonological factors that go into repetition tasks. Other factors may include phonological segmentation, phonological blending, and assembly of articulatory motor programs.

^2^It is noteworthy that electronic writing in the social media, such as Facebook and SMS, are often conducted in the local spoken dialects using either the Roman alphabet along with a few numerals representing some of the unique Arabic sounds, or a modified version of the Arabic alphabet. The use of this variety for reading and writing in the electronic media emerges naturally among users and no formal instruction in using it is provided.

## Appendix A

**Table 6:** Screening tasks: summary statistics and repeated measure anova results.

	**SLI-SK** **(*N* = 25)**	**TD-SK** **(*N* = 25)**	**SLI-G1** **(*N* = 25)**	**TD-G1** **(*N* = 25)**	***F*_(1, 96)_** **(Grade)**	***F*_(1, 96)_** **(Group)**	***F*_(1, 96)_** **(Group^*^Grade)**
RAN for Colors (s)	2.10^ac^ (0.80)	1.34^b^ (0.24)	1.52^d^ (0.44)	1.16 (0.25)	14.80^***^ $\eta_{p}^{2}$ = 0.13	32.49^***^ $\eta_{p}^{2}$ = 0.25	4.11^*^ $\eta_{p}^{2}$ = 0.04
Forward Digit Span	10.88^ac^ (5.78)	19.60^b^ (4.72)	18.84^d^ (5.50)	21.72 (4.07)	24.79^***^ $\eta_{p}^{2}$ = 0.21	32.83^***^ $\eta_{p}^{2}$ = 0.26	8.32^**^ $\eta_{p}^{2}$ = 0.08
Word Articulation	82.52^ac^ (9.59)	99.65^b^ (1.20)	99.57^d^ (1.54)	100 (0.00)	78.96^***^ $\eta_{p}^{2}$ = 0.45	80.55^***^ $\eta_{p}^{2}$ = 0.46	72.77^***^ $\eta_{p}^{2}$ = 0.43
Expressive Vocabulary	51.89^ae^ (10.82)	76.60^b^ (4.72)	65.26^cf^ (6.35)	75.23^d^ (6.98)	14.40^***^ $\eta_{p}^{2}$ = 0.13	120.28^***^ $\eta_{p}^{2}$ = 0.56	21.72^**^ $\eta_{p}^{2}$ = 0.19
Sentence Completion	26.00^ag^ (11.60)	57.41^bc^ (7.79)	55.53^eg^ (10.16)	71.65^df^ (6.46)	140.73^***^ $\eta_{p}^{2}$ = 0.59	165.98^***^ $\eta_{p}^{2}$ = 0.63	17.19^***^ $\eta_{p}^{2}$ = 0.15
Sentence Repetition	26.21^ae^ (18.46)	83.35^b^ (6.679)	54.00^cf^ (15.90)	90.32^d^ (4.56)	32.64^***^ $\eta_{p}^{2}$ = 0.25	99.63^***^ $\eta_{p}^{2}$ = 0.51	11.38^***^ $\eta_{p}^{2}$ = 0.11
Non-word Repetition	37.88^ae^ (18.33)	80.47^b^ (15.77)	60.59^cf^ (16.66)	85.88^d^ (7.35)	21.57^***^ $\eta_{p}^{2}$ = 0.18	125.71^***^ $\eta_{p}^{2}$ = 0.57	8.16^**^ $\eta_{p}^{2}$ = 0.08
Non-word Discrimination	15.30^ag^ (22.01)	82.88^bc^ (9.89)	59.93^eh^ (19.80)	99.65^df^ (1.24)	96.60^***^ $\eta_{p}^{2}$ = 0.50	294.96^***^ $\eta_{p}^{2}$ = 0.75	19.89^**^ $\eta_{p}^{2}$ = 0.17

* *p < 0.05*,

** *p < 0.01*,

*** *p < 0.001*.

*Superscripted letters indicate Bonferroni Pairwise test results*.
